# Serum ferritin is a good indicator for predicting the efficacy of adult HLH induction therapy

**DOI:** 10.1080/07853890.2022.2027513

**Published:** 2022-02-03

**Authors:** Zhengjie Hua, Lingbo He, Ruoxi Zhang, Menghan Liu, Zhao Wang, Yini Wang

**Affiliations:** a Department of Hematology, Capital Medical University Affiliated Beijing Friendship Hospital, Beijing, China; b Department of General Medicine, Capital Medical University Affiliated Beijing Friendship Hospital, Beijing, China

**Keywords:** Adults, haemophagocytic lymphohistiocytosis, induction therapy, ferritin, efficacy

## Abstract

**Background: **Hemophagocytic lymphohistiocytosis (HLH) is a rare clinical syndrome with a high mortality rate. There is no biomarker to predict the early therapeutic response.

**Objective: **Our study explores the significance of serum ferritin in predicting the response of induction therapy.

**Methods:** We retrospectively analyzed the clinical data of 102 adult patients with HLH admitted to our hospital. These patients received HLH-94 regimen for induction therapy. The patients were divided into remission group and non-remission group according to the response of induction therapy.

**Results:** Ferritin values between 1–4 weeks post induction were predictive of remission (*p*<.05), which were higher in the non-remission group than in the remission group. Ferritin obtained 2 weeks post-induction had the highest ROC for predicting remission, with a cut-off value of 1188.5 µg/L. And patients with ferritin lower than 1188.5 µg/L had better response of induction therapy.

**Conclusion:** Our study suggests that serum ferritin is a good indicator to predict the efficacy of induction therapy for adult HLH.

**KEY MESSAGES**
- Serum ferritin is a good indicator for predicting the efficacy of adult HLH induction therapy.
- Serum ferritin two weeks after treatment may be a better indicator to judge the early curative effect.
- Serum ferritin after treatment also had a predictive significance for the survival of HLH.

## Introduction

Hemophagocytic lymphohistiocytosis (HLH) is a hyperinflammatory syndrome caused by abnormally activated macrophages and cytotoxic T cells [1], which can be classified as primary and secondary according to aetiology. Primary HLH is predominantly found in children, caused by genetic defects of proteins involved in cytotoxic function in T lymphocytes and natural killer NK cells. Secondary HLH is mainly seen in adults and is often triggered by infection, malignant tumours, auto-inflammatory/autoimmune diseases or other unexplained disorders. Failure to receive timely and appropriate treatment will result in a mortality rate of 50–75% [2]. Studies have shown that the response of induction therapy is the most relevant factor affecting the overall survival of patients with HLH [3].

Serum ferritin is an intracellular storage protein, which is mainly found in macrophages [4]. The increase may be related to the release of tissue macrophages, and ferritin is a marker of macrophage activation, which may reflect the immune activation state of the body [5]. Studies have shown that a significantly elevated serum ferritin is a common feature of all types of HLH, and the HLH-2004 guidelines have included “Ferritin ≥500 µg/L” as one of the eight diagnostic criteria for HLH. It has been found that serum ferritin, in addition to its diagnostic value, may be an important indicator of the efficacy of HLH treatment [6]. We mainly explored ferritin as a marker of induction therapeutic response in HLH.

## Methods

### Patients

**Figure 1.**
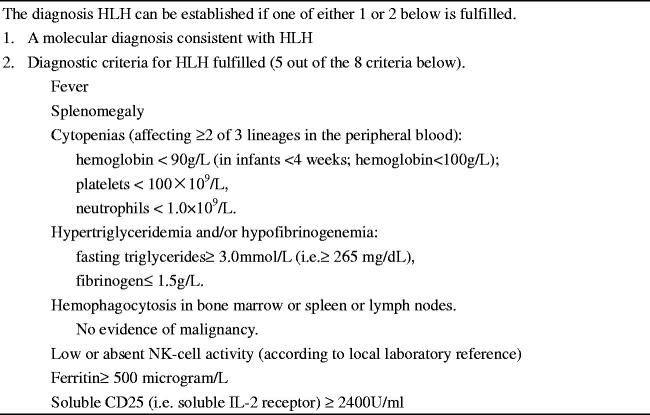
HLH-2004 diagnostic criteria.

**Figure 2.**
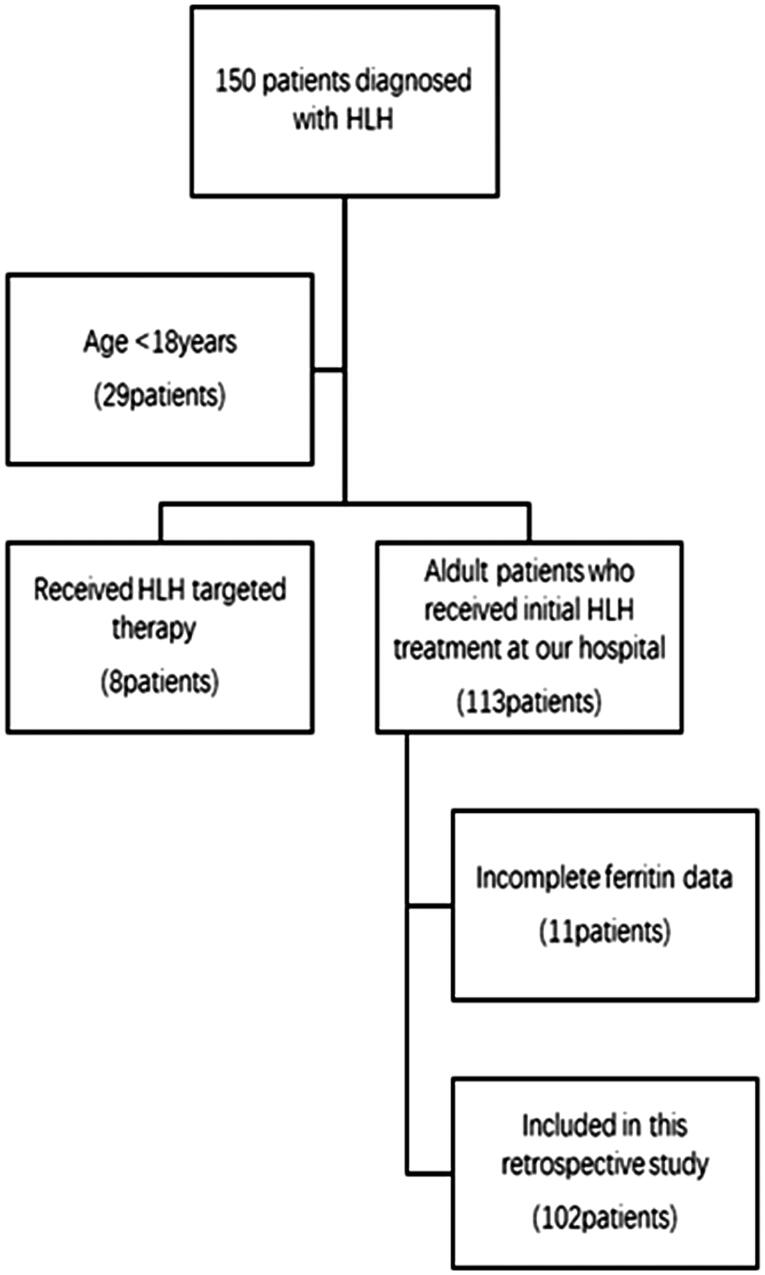
Diagram of enrolled patients.

This is a retrospective study with 150 patients who were admitted to the hospital with a preliminary diagnosis of HLH between January and December 2017. Inclusion criteria: (1) Met the HLH-2004 diagnostic criteria (Figure 1) [1]; (2) Age ≥18 years; (3) Patients who received initial HLH treatment at our hospital. Exclusion criteria: (1) Age <18 years; (2) Received HLH targeted therapy; (3) Incomplete ferritin data. We finally analysed 102 adult HLH patients who received initial induction therapy at our institution (Figure 2). The follow-up period was calculated from the date of admission with a diagnosis of HLH to two years of follow-up or the time of death due to any reason. This research was in line with the Declaration of Helsinki and approved by the Ethics Committee at Beijing Friendship Hospital, Capital Medical University at July 2019.

### Parameters associated with HLH

We analysed retrospectively the patients' general conditions (including: age, gender, race), aetiology, baseline biochemical parameters, all parameters included in the HLH-2004 Diagnostic Criteria (fever, splenomegaly, hemophagocytosis in bone marrow, cytopenias affecting at least two spectrums, triglycerides, fibrinogen, ferritin, natural killer (NK) cytotoxic activity, sCD25, ferritin) and induction therapy regimens. The NK cell activity assay applied in this paper is a patented technology of our research centre. The type of ferritin assay employed at the Hospital is Beckman Coulter. The normal reference value of SF is 10–120 µg/L for females and 20–300 µg/L for males. We mainly analysed the predictive effect of ferritin on the response of the induction treatment.

### Treatments

All patients enrolled in this study received HLH-94 strategies for induction therapy, which included dexamethasone, etoposide, cyclosporine, and intrathecal methotrexate and dexamethasone. We made age-related adjustments for the use of etoposide in adults due to the lower need for and tolerance of etoposide (75–100 mg/m^2^ in patients aged 18–39 years; 50–75 mg/m^2^ in patients aged 40 years and older). Dexamethasone, 10 mg/m^2^/d for one to two weeks, 5 mg/m^2^/d for three to four weeks, 2.5 mg/m^2^/d for five to six weeks, 1.25 mg/m^2^/d for seven weeks, reduced to discontinuation for eight weeks. We adjusted the dose and duration of dosing based on individual patient characteristics. And we performed an assessment for initial induction therapy response after two to four weeks of induction therapy with the HLH94 regimen. We continued the current HLH94 regimen of induction therapy for eight weeks for patients who achieved remission. For unrelieved patients, we switched to a DEP regimen for induction therapy, which includes liposomal doxorubicin treatment combined with etoposide and methylprednisolone.

### Response assessment

Efficacy evaluation refers to the assessment of response based on patient symptoms and laboratory indicators after eight weeks of induction therapy. In this study, the evaluation criteria for efficacy was mainly based on the HLH Chinese expert consensus [7]. The main indicators of efficacy evaluation included sCD25, ferritin, blood cell count, triacylglycerol, hemophagocytosis in bone marrow and level of consciousness (in the presence of CNS-HLH). Complete response (CR): all of the above indicators returned to normal range. Partial response (PR): 25% or more improvement in ≥2 symptoms/laboratory indicators. Non-response: no improvement in clinical symptoms and the above indicators. Patients were divided into remission group (complete response CR and partial response PR) and non-remission group (non-responders after treatment) according to the response to induction therapy.

### Statistical analysis

All data were processed by SPSS 26.0 statistical software. Measurement data conforming to normal distribution were represented by mean ± standard deviation, and non-normal distribution was represented by median and percentile. Independent samples t-test, Mann–Whitney U test and Chi-Square test were employed to make a comparison between groups for normally distributed variables, non-normally distributed variables and categorical variables, respectively; ROC curve was applied to evaluate the value of serum ferritin in predicting the response of induction treatment and the best cut-off points for ferritin; Kaplan-Meier estimator test was used for response and survival analysis. Multivariate analysis was conducted using logistic regression. Two-tailed *p* < .05 was considered statistically significant.

## Results

### Patients' characteristics

Out of 102 patients, aetiologies of HLH were categorized as infectious diseases (*N* = 46), malignancies (*N* = 28), autoimmune disorders (*N* = 9), primary HLH (*N* = 6) and no identified underlying disorder (*N* = 24) (Figure 2). Patients were divided into remission group (*n* = 53) and non-remission group (*n* = 49) based on the response of induction treatment. All patient races were Chinese, and fever was observed in all patients. A comparison was made between the two groups by non-parametric test. And results showed that compared with remission group, the levels of sCD25 (*p* = .035), direct bilirubin (*p* = .034) and urea (*p* = .030) upon admission were significantly higher and the values of platelets (*p* = .003) were significantly lower in non-remission group. It also showed there was significant difference in etiology. Regarding to race, age, gender, clinical signs, laboratory variables (haemophagocytosis, TG, FIB, NK% activity, admission ferritin, WBC, NEUT, HB, ALT, AST, LDH, HDL, LDL, ALB, ALP, UA and Ca2+), there was no significant difference between the two groups (all *p* > .05) (Table 1). In order to exclude the interaction between the factors, we conducted a multifactorial analysis of the five indicators mentioned above. Results showed that there was no significant difference in the above five indicators between the two groups (all *p* > .05) (Table 2).

**Table 1.** General information and laboratory indicators of the included patients.

	Remission	Non-remission	*p*
Median age (years)	36 (26–55)	44 (29–53)	.406
Race			
(All Chinese)	53	49	
Gender	53	49	.314
(Male/female)	25/28	28/21	
Aetiology	53	49	.025
Infectious diseases	20/53	26/49	
Malignancies	11/53	17/49	
Autoimmune disorders	7/53	2/49	
Primary HLH	3/53	3/53	
No identified	12/53	12/53	
Fever (all patients)	53	49	
Splenomegaly	53	49	.363
(N/Y)	15/38	18/31	
Haemophagocytosis	53	49	.254
(N/Y)	11/42	15/34	
TG (mmol/L)	2.32 (1.6–3)	2.4 (1.8–3.6)	.606
FIB (g/L)	2.04 (1.4–3.8)	1.57 (1.08–2.36)	.074
NK % activity	15.13% (13%–16.56%)	14% (12.84%–17.78%)	.616
sCD25 (pg/ml)	13682 (4775–33881)	25982 (14414–39578)	.035
Admission ferritin (μg/L)	1608 (881–7256)	2256 (1156–12481)	.24
WBC (×10^9^/L)	3.60 (1.94–6.75)	3.37 (1.26–5.28)	.095
NEUT (×10^9^/L)	1.79 (1.11–4.67)	1.69 (0.72–3.33)	.134
HB (g/L)	85 (72.5–106.5)	84 (68–108)	.112
PLT (×10^9^/L)	105 (62–222)	61 (22.5–98.5)	.003
ALT (U/L)	76 (27.5–154.5)	52 (38.5–106.3)	.684
AST (U/L)	57 (22–101)	73.5 (30.8–162)	.327
LDH (U/L)	428 (322.5–629.5)	418.5 (277.8–873.8)	.714
DBIL (µmol/L)	4.82 (2.54–9.28)	7.24 (3.78–21.65)	.034
HDL (mmol/L)	0.66 (0.47–1.1)	0.68 (0.35–1.06)	.342
LDL (mmol/L)	2.27 (1.71–2.91)	1.94 (1.34–2.89)	.198
ALB (g/L)	30.76 ± 5.7	29.23 ± 5.11	.49
Urea (mmol/L)	4.24 (3.18–6.65)	5.72 (4.38–6.74)	.03
ALP (U/L)	136 (81–271.5)	134.5 (87–446.5)	.522
UA (µmol/L)	220 ± 97	235 ± 85	.464
Ca2+ (mmol/L)	2.07 ± 0.169	2.02 ± 0.171	.323
Induction treatment (all received HLH-94 regimen)	53	49	
Treatment response at eight weeks	53	49	

TG: triglyceride; FIB: fibrinogen; WBC: white blood cell; NEUT: neutrophile granulocyte; HB: haemoglobin; PLT: platelets; ALT: alanine aminotransferase; AST: aspartate aminotransferase; LDH: lactate dehydrogenase; DBIL: direct bilirubin; HDL: high-density lipoprotein; LDL: low density lipoprotein; ALB: albumin; ALP: alkaline phosphatase; UA: uric acid; Ca2+: calcium.

**Table 2.** Multivariate analyses of factors which affect the response of induction therapy.

Variables	Multivariate analysis		
	OR	95%CI	*p*
PLT (×10^9^/L)	1.004	0.997–1.010	.250
DBIL (µmol/L)	0.983	0.964–1.004	.108
Urea (mmol/L)	0.869	0.701–1.077	.200
sCD25 (pg/ml)	1.000	1.0–1.0	.993
Aetiology	0.776	0.060–10.096	.847

PLT: platelets; DBIL: direct bilirubin.

### Comparison of ferritin between the two groups

Through the independent sample non-parametric test of data from the two groups, it can be calculated that there were significant differences in ferritin levels between the two groups at one to four weeks post induction, and the serum ferritin level of patients in the non-remission group was significantly higher than that in the remission group (*p* = .013, *p* = .004, *p* = .005, *p* = .002). There was no statistical difference between the two groups in ferritin levels before treatment (*p* = .526) (Table 3).

**Table 3.** Ferritin and response to induction therapy.

	Remission	Non-remission	*p*
Before treatment (μg/L)	3652 (976–8362)	2722 (1218–15007)	.526
One week after treatment (μg/L)	1554 (850–3801)	2789 (1416–11442)	.013
Two weeks after treatment (μg/L)	903 (498–1947)	1720 (1464–3564)	.004
Three weeks after treatment (μg/L)	755 (549–1734)	1883 (1385–3717)	.005
Four weeks after treatment (μg/L)	631 (273–1493)	2205 (919–3749)	.002

### Relationship among ferritin with induction response

**Figure 3.**
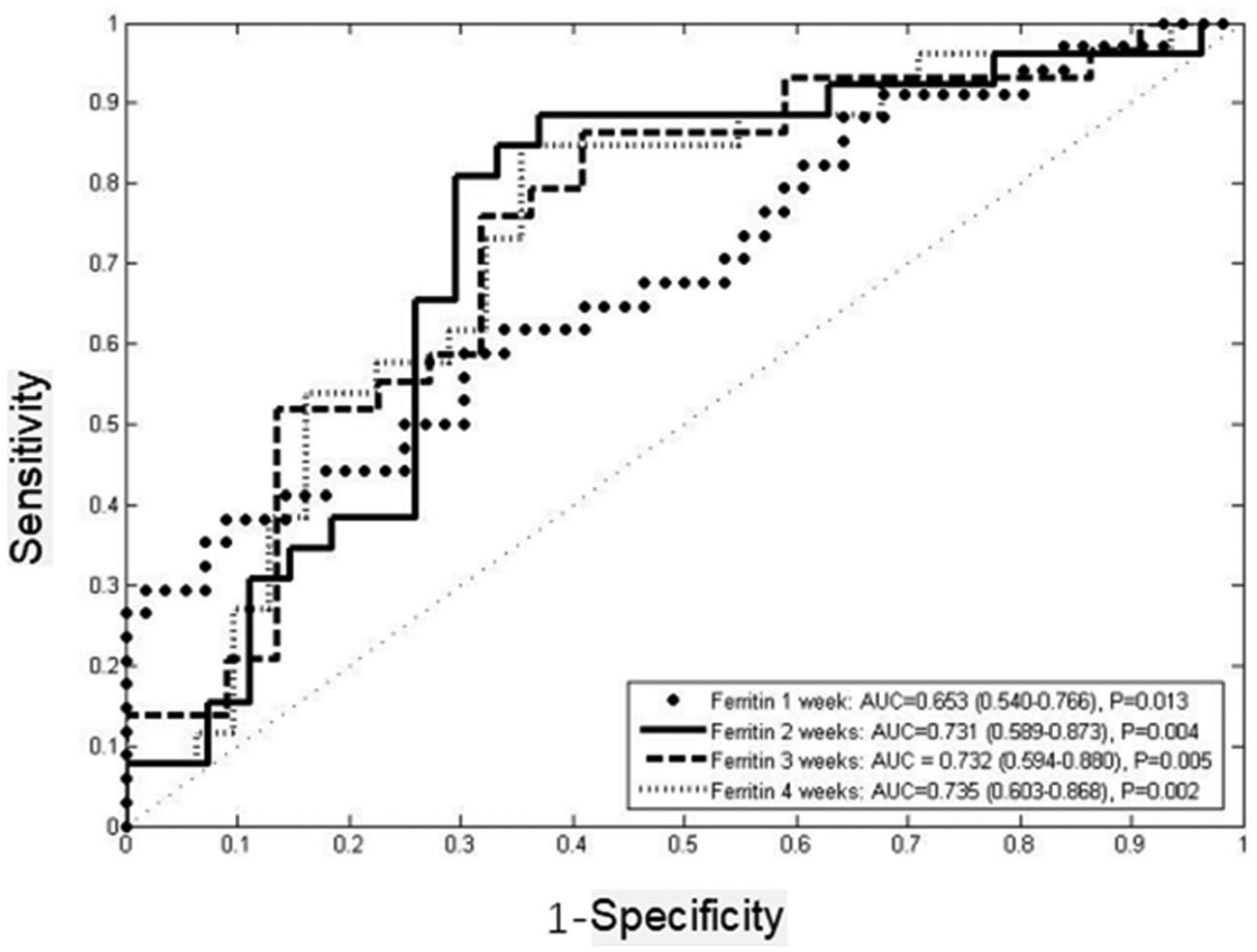
Comparison of AUC of ferritin in predicting induction response.

ROC curves were employed to explore the ability of ferritin at one to four weeks after induction treatment in predicting the response of induction treatment (Figure 3). The results showed that the area under the curve of ferritin at one to four weeks after treatment was greater than 0.5. Ferritin at two weeks after treatment was the first indicator to predict the response of the induction treatment with 88.5% sensitivity and 63% specificity (AUC = 0.731, 95%CI 0.589–0.873, *p* = .004), and the best cut-off value was 1188.5 µg/L. Ferritin at three weeks was the second indicator to predict the induction response with 85.7% sensitivity and 59.1% specificity (AUC = 0.732, 95%CI 0.594–0.880, *p* = .005), and the best cut-off value was 1149.0 µg/L. Ferritin at four weeks was the third indicator to predict the induction response with 84.6% sensitivity and 64.5% specificity (AUC = 0.735, 95%CI 0.603–0.868, *p* = .002). Ferritin at one week after treatment had the lowest AUC (AUC = 0.653, 95%CI 0.540–0.766, *p* = .013) with 57.8% sensitivity and 68.9% specificity, and the best cut-off value was 2602 µg/L (Tables 4 and 5).

**Table 4.** Ferritin for predicting induction response.

	AUC	*p*	Sensitivity	Specificity	Cut-off value (μg/L)
Ferritin one week after treatment	0.653 (0.540–0.766)	.013	0.578	0.689	2602.0
Ferritin two weeks after treatment	0.731 (0.589–0.873)	.004	0.885	0.63	1188.5
Ferritin three weeks after treatment	0.732 (0.594–0.880)	.005	0.857	0.591	1149.0
Ferritin four weeks after treatment	0.735 (0.603–0.868)	.002	0.846	0.645	846.5

**Table 5.** Ferritin for predicting survival.

	AUC	*p*	95% CI	Cut-off value (μg/L）	Sensitivity	Specificity
One week	0.545	.508	0.420–0.670			
Two weeks	0.584	.348	0.404–0.764			
Three weeks	0.743	.009	0.590–0.896	1442.5	0.684	0.769
Four weeks	0.701	.017	0.537–0.866	803.0	0.744	0.706

**Figure 4.**
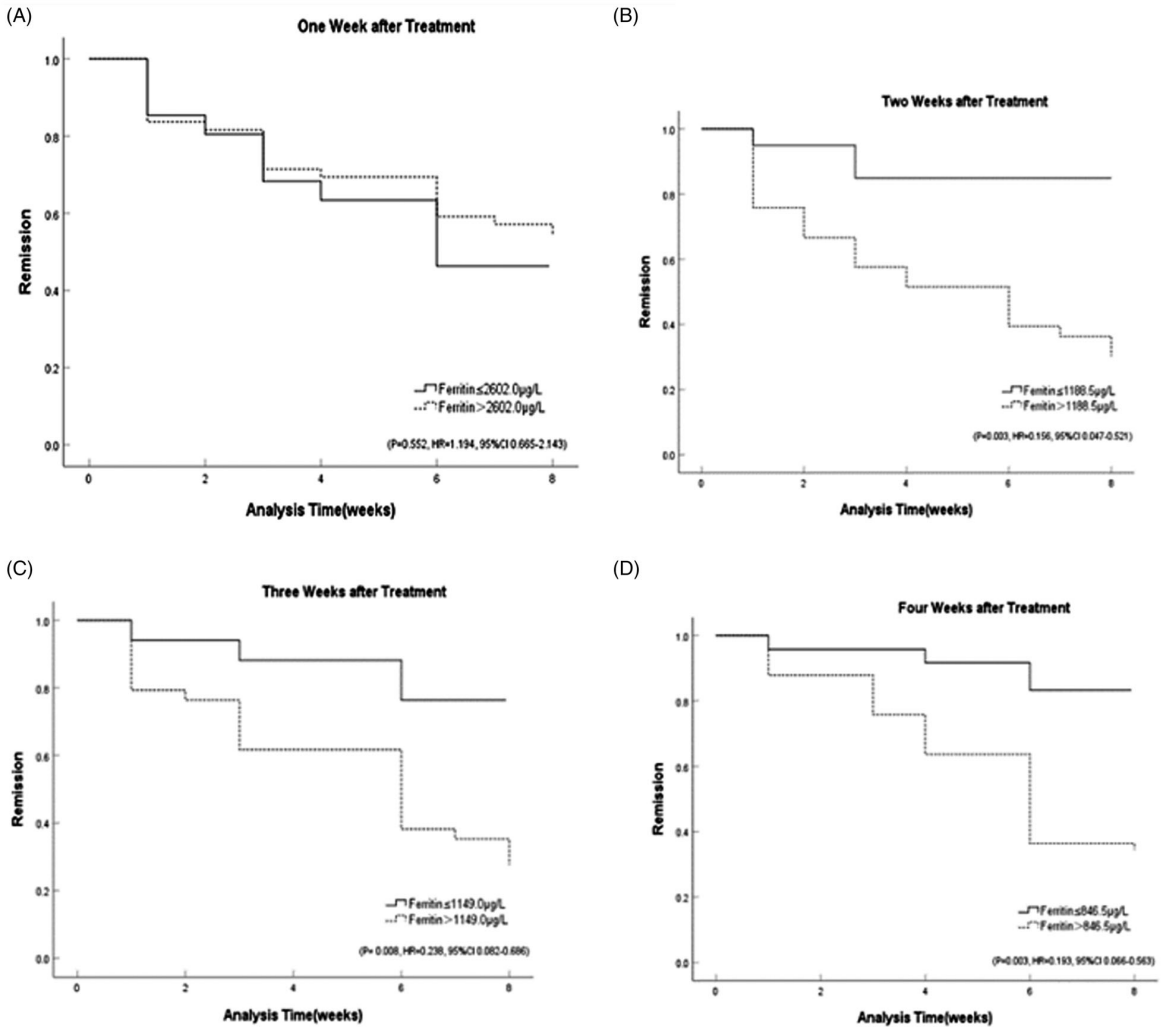
Response stratified by ferritin cut-off post-therapy initiation at: (A) one week (*p* = 0.552, HR = 1.194, 95%CI 0.665–2.143); (B) two weeks (*p* = 0.003, HR = 0.156, 95%CI 0.047–0.521); (C) three weeks (*p* = 0.008, HR = 0.238, 95%CI 0.082–0.686); (D) four weeks (*p* = 0.003, HR = 0.193, 95%CI 0.066–0.563).

**Figure 5.**
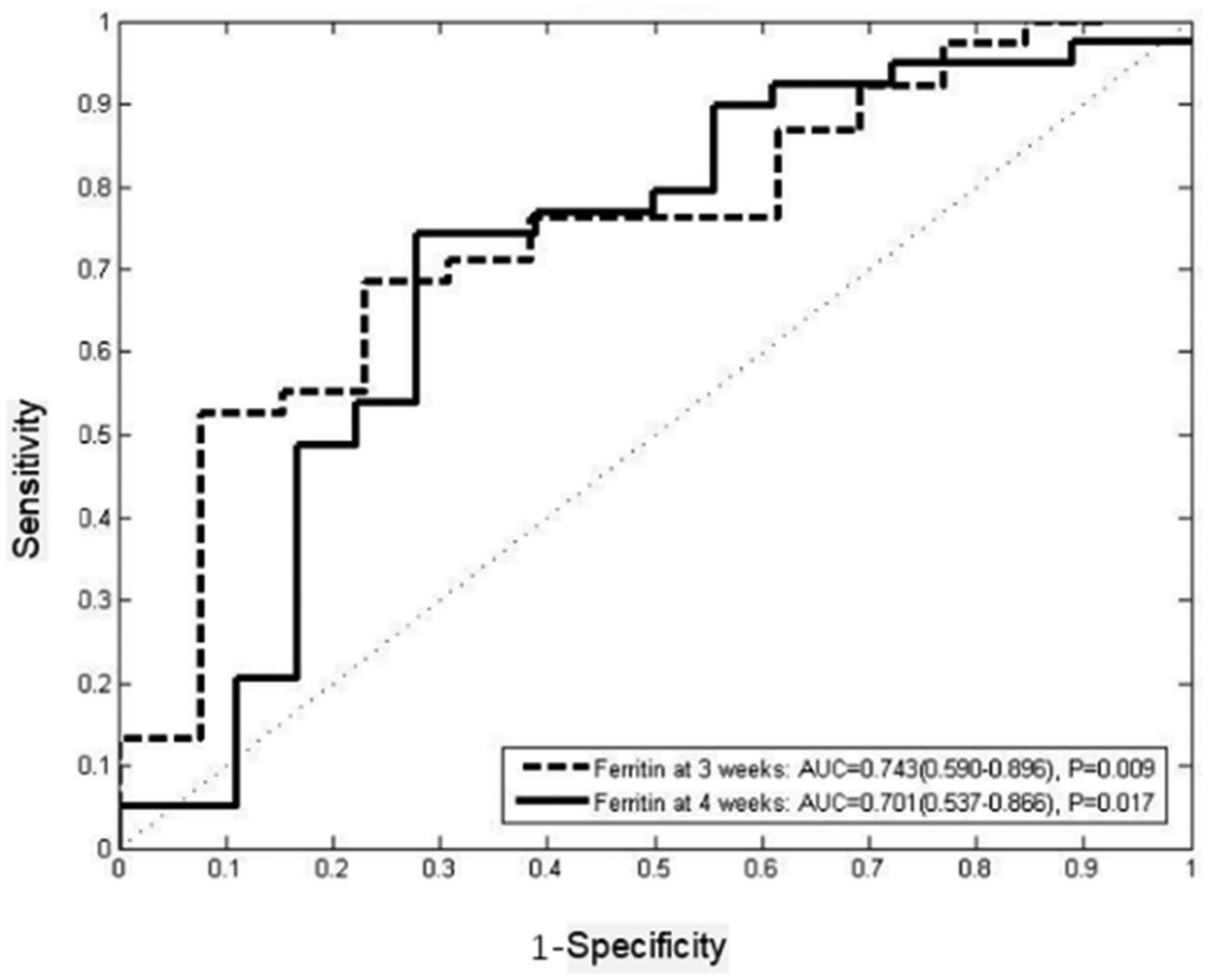
Comparison of AUC of ferritin in predicting survival.

The Kaplan-Meier method was applied to analyse the response to induction therapy in patients with different stratification of ferritin values. The results showed that HLH patients with ferritin values less than 1188.5 µg/L after two weeks of treatment had a better response at the eight-week evaluation (*p* = .003, HR = 0.156, 95%CI 0.047–0.521). Ferritin at three weeks less than 1149.0 µg/L after induction treatment (*p* = .008, HR = 0.238, 95%CI 0.082–0.686) or ferritin at four weeks less than 846.5 µg/L (*p* = .003, HR = 0.193, 95%CI 0.066–0.563) were also significantly associated with response, while results showed that ferritin at one week after treatment was not statistically significant to predict the response of induction (*p* = .552, HR = 1.194, 95%CI 0.665–2.143) (Figures 4 and 5).

### Survival analysis of ferritin and long-term outcome

**Figure 6.**
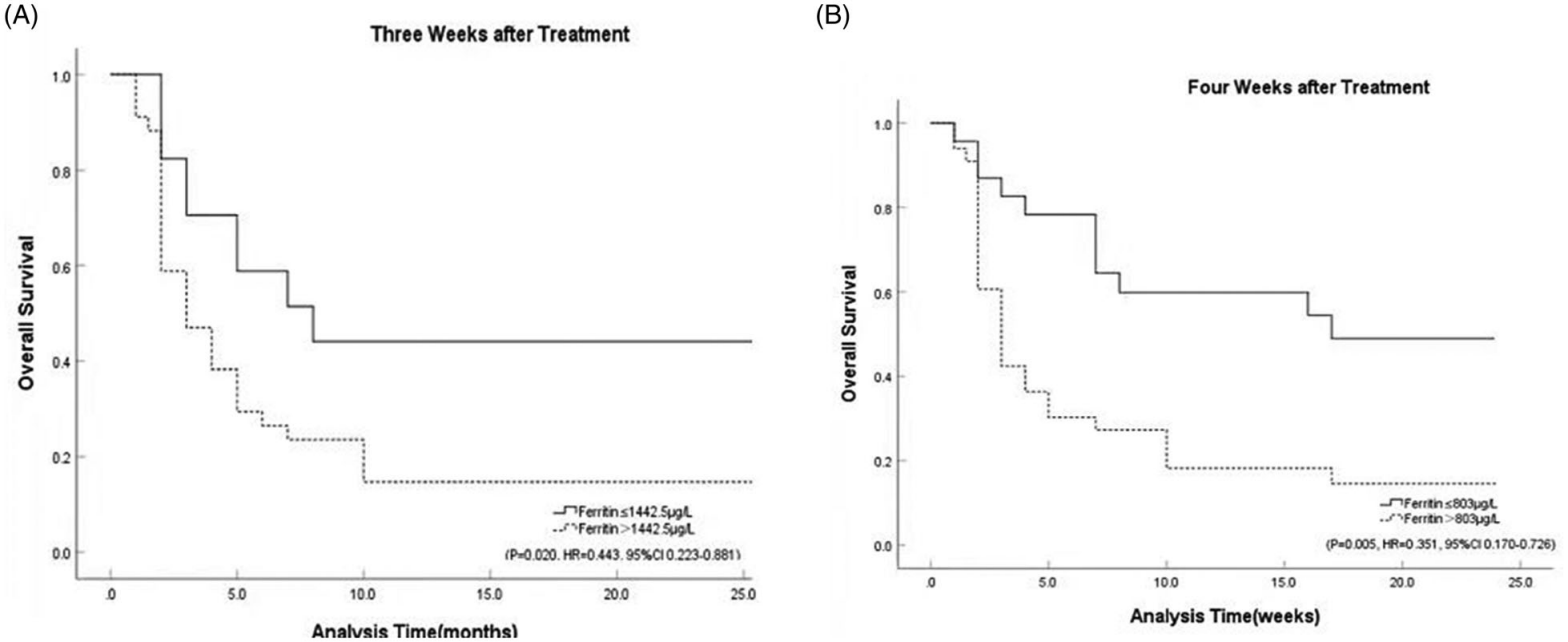
The survival curve for ferritin. (A) The survival curve for ferritin at 3 weeks after treatment (*p* = 0.020, HR = 0.443, 95%CI 0.223–0.881). (B) The survival curve for ferritin at 4 weeks after treatment (*p* = 0.005, HR = 0.351, 95%CI 0.170–0.726).

We followed the patients for two years and analysed the correlation among ferritin values after induction therapy with the overall survival. Results showed that ferritin levels at three weeks (*p* = .009) and four weeks (*p* = .017) were significantly associated with long-term outcome. While the results indicated that the ferritin at one week (*p* = .508) and two weeks (*p* = .348) after treatment might did not predict survival. ROC curves were employed to analysed the ability of ferritin at three and four weeks after induction therapy to predict two-year survival. The results showed that ferritin at three weeks after treatment was the first indicator to predict the overall survival with 68.4% sensitivity and 76.9% specificity (AUC = 0.743, 95%CI 0.590–0.896, *p* = .009), and the best cut-off value was 1442.5 µg/L. Ferritin at four weeks after treatment was the second indicator to predict the overall survival with 74.4% sensitivity and 70.6% specificity (AUC = 0.701, 95%CI 0.537–0.866, *p* = .017), and the best cut-off value was 803 µg/L. Kaplan–Meier curve showed that ferritin at three (*p* = .020, HR = 0.443, 95%CI 0.223–0.881) and four weeks after treatment (*p* = .005, HR = 0.351, 95%CI 0.170–0.726) might have a predictive effect on long-term outcome (Figure 6).

## Discussion

HLH is a high-inflammatory factor storm syndrome characterized by an extreme increase in pro-inflammatory cytokines. The early symptoms are atypical, and the diagnosis is difficult. However, the disease progresses rapidly, and many patients die rapidly due to seeking medical treatment too late or unclear diagnosis. Thus, early diagnosis and treatment of HLH are very important. The reactants in the acute phase of HLH are often elevated, and the most relevant one is serum ferritin [8]. Ferritin is an acute-phase reactive protein that regulates the homeostasis and storage of iron. It mainly exists in reticuloendothelial cells. The content in macrophages is the highest [4]. And the extensive activation of macrophages may be responsible for the significant increase of ferritin in HLH [9]. Studies have found that increased serum ferritin is a clinical parameter of iron overload, inflammation, tumour burden and liver disease [10]. In adults, the most common condition associated with elevated ferritin is haematological malignancies, followed by liver failure, HLH and other common causes including infection, kidney failure, chronic blood transfusion and haemoglobinopathy. Studies have shown that increased ferritin has no significant specificity for the diagnosis of HLH in adults [11], and not all hyperferritinemia patients have HLH. Therefore, clinical manifestations and other laboratory indicators should be combined to identify the disease in diagnosis.

The current diagnostic criteria for HLH 2004 in adults set “Ferritin ≥500 µg/L” as one of the diagnostic criteria. This result is mainly based on the threshold value for the diagnosis of HLH in children. The threshold value of ferritin is different in patients of different ages, and ferritin diagnostic thresholds have not been verified in adult HLH [12]; therefore, ferritin in diagnosis and predicting the efficacy of adult HLH is worthy of further study. Currently, most studies focus on its ability to diagnose HLH. Research has found that setting the threshold value of ferritin at 2000 µg/L will have a higher diagnostic value for adult HLH [13]. Some scholars also believe that ferritin can be used as a negative evaluation index for HLH. Most studies on ferritin and the prognosis of adult HLH have evaluated its relationship with overall survival. The results indicate that ferritin can be used as an independent prognostic indicator for adult HLH [6]. When the ferritin decline rate is less than 50%, the mortality rate of patients is 17 times higher than that when it is reduced by 96% or more [14]. However, ferritin is an acute-phase reactive protein, and the relationship between ferritin and early induction response has not been fully studied. In a multivariate analysis, the researchers found that the response of eight-week treatment was the most relevant factor affecting the overall survival of HLH [3] and was a good predictor of overall survival. Therefore, the response to induction therapy is important throughout the course of HLH and deserves our attention. Early prediction of the response of induction therapy facilitates clinicians to assess the sensitivity of patients to the current treatment and adjust the treatment plan in time. In the assessment of patients with poor induction therapy response, ferritin is a simple and intuitive laboratory indicator of the disease status of HLH compared to other indicators such as sCD25 and NK cell activity [15]. Our study focussed on the predictive value of ferritin for response to HLH induction therapy.

This article compared the correlation among general conditions and various laboratory indicators at admission with the response of induction treatment by chi-square test and independent samples non-parametric test. We found that statistically significant increases in sCD25, direct bilirubin and urea levels and statistically significant decreases in platelet at admission in the non-remitting group compared to the remitting group [23]. We also found significant differences in aetiology. This conclusion is supported by the results of Fardet et al. [16,17]. However, when the multifactorial analysis overcame the interactions between the indicators, it showed that there were no independent risk factors for HLH as described above. Predicting induction response by a single static indicator before treatment seems inadequate, considering the interrelated laboratory indicators and the complex course of HLH influenced by multiple factors. Some studies have found that whether patients received an appropriate treatment regimen is an important factor influencing response [18]. Therefore, the ferritin after induction treatment has clinical significance for predicting the response of induction therapy.

We found that ferritin two weeks after treatment had some predictive value for response to induction therapy. Studies have shown that ferritin may be a useful indicator of disease activity and prognosis in adult HLH patients [19]. In this study, we monitored ferritin at admission and for four consecutive weeks after induction treatment in patients with HLH. The results showed no significant correlation between pre-treatment ferritin and response during induction therapy, which was supported by the findings of Arca et al. [16]. Ferritin levels one to four weeks after treatment were correlated with the response of induction therapy. Patients in the remission group had significantly lower ferritin levels than those in the non-remission group. The findings of our study are supported by the results of Zhou et al. [6,20]. Notably, the ROC curves of ferritin in predicting induction response suggests that ferritin at two to four weeks after treatment can be used as an indicator of response to induction therapy. The Kaplan–Meier method of ferritin in predicting induction response also supports this idea. Therefore, we tend to believe that ferritin levels at two to four weeks after treatment seem to be a good predictor of early response. Regarding timing of salvage therapy, ferritin two-week post-treatment for predicting response of induction therapy can help physicians assess disease earlier by contrast, and patients with two-week post-treatment ferritin levels below 1188.5 µg/L have an increased response during induction therapy. There is no international standard for judging the timing of salvage therapy, and most scholars believe that salvage therapy can be started if there is no good response after two to three weeks of initial HLH treatment. Our study found that ferritin two to four weeks after treatment can be a better predictor of early response to induction therapy. It is recommended that monitoring serum ferritin two weeks after treatment in HLH patients will help to predict the response to induction therapy earlier, which in turn will facilitate physicians to initiate salvage therapy in a timely manner.

In contrast to previous studies, this paper focuses on the predictive value of ferritin for induction response in patients with HLH. Previous investigators have discussed the relationship between ferritin and the prognosis of patients at six months, one year and two years after treatment and conclude that serum ferritin is a good predictor of survival [21,22]. Our study also analysed the predictive role of ferritin on survival, and the results showed significant differences in survival among patients with different ferritin stratification at three to four weeks after induction therapy. Therefore, we conclude that ferritin at three to four weeks after induction treatment might have predictive significance for the long-term outcome of HLH. However, during the long course of HLH, the survival is affected by various factors, such as infection, progression of HLH relapse, multi-organ dysfunction and progression of secondary HLH primary disease. On the one hand, ferritin is not only elevated in HLH patients, its elevation can be seen in patients with infection, inflammation and liver and kidney failure. Ferritin used for predicting the early induction response is relatively less influenced by the above factors. On the other hand, ferritin is an acute-phase response protein, which has a better value in reflecting the acute phase of the disease. Therefore, although it has some value in predicting survival, we mainly focus on the significance of ferritin for predicting the response of induction treatment.

### Limitations

Due to the retrospective nature of the study, we had to face a certain degree of missing data from clinical and laboratory investigations or patients who were not followed up. Although the number of patients was sufficient for the final analysis, multicentre prospective randomized clinical trials with larger sample size are needed to further verify our results. In addition, individual differences in HLH patients and disease management among physicians vary widely.

## Conclusions

Ferritin post-induction may be a predictor of induction response and long-term outcome. Ferritin values two to four weeks after induction therapy appears to be a useful marker for predicting induction response. And attaining a ferritin below 1188 µg/L at two weeks post-induction is an indicator of a better induction response. Ferritin helps to predict the response to induction therapy early and helps clinicians to initiate salvage therapy in a timely manner.

## Data Availability

The datasets used during the current study are available from the corresponding author on request.
